# Precision of morphogen gradients in neural tube development

**DOI:** 10.1038/s41467-022-28834-3

**Published:** 2022-03-03

**Authors:** Roman Vetter, Dagmar Iber

**Affiliations:** 1 Department of Biosystems Science and Engineering, ETH Zürich, Mattenstrasse 26, 4058 Basel, Switzerland; 2 Swiss Institute of Bioinformatics, Mattenstrasse 26, 4058 Basel, Switzerland

**Keywords:** Computational models, Computational biophysics, Developmental neurogenesis, Pattern formation

## Abstract

Morphogen gradients encode positional information during development. How high patterning precision is achieved despite natural variation in both the morphogen gradients and in the readout process, is still largely elusive. Here, we show that the positional error of gradients in the mouse neural tube has previously been overestimated, and that the reported accuracy of the central progenitor domain boundaries in the mouse neural tube can be achieved with a single gradient, rather than requiring the simultaneous readout of opposing gradients. Consistently and independently, numerical simulations based on measured molecular noise levels likewise result in lower gradient variabilities than reported. Finally, we show that the patterning mechanism yields progenitor cell numbers with even greater precision than boundary positions, as gradient amplitude changes do not affect interior progenitor domain sizes. We conclude that single gradients can yield the observed developmental precision, which provides prospects for tissue engineering.

## Abstract (web summary)

Morphogen gradients encode positional information during development. Here the authors use theory and simulations to suggest a positional accuracy of single gradients that directly explains the observed precision of progenitor domain boundaries.

## Introduction

**Fig. 1:**
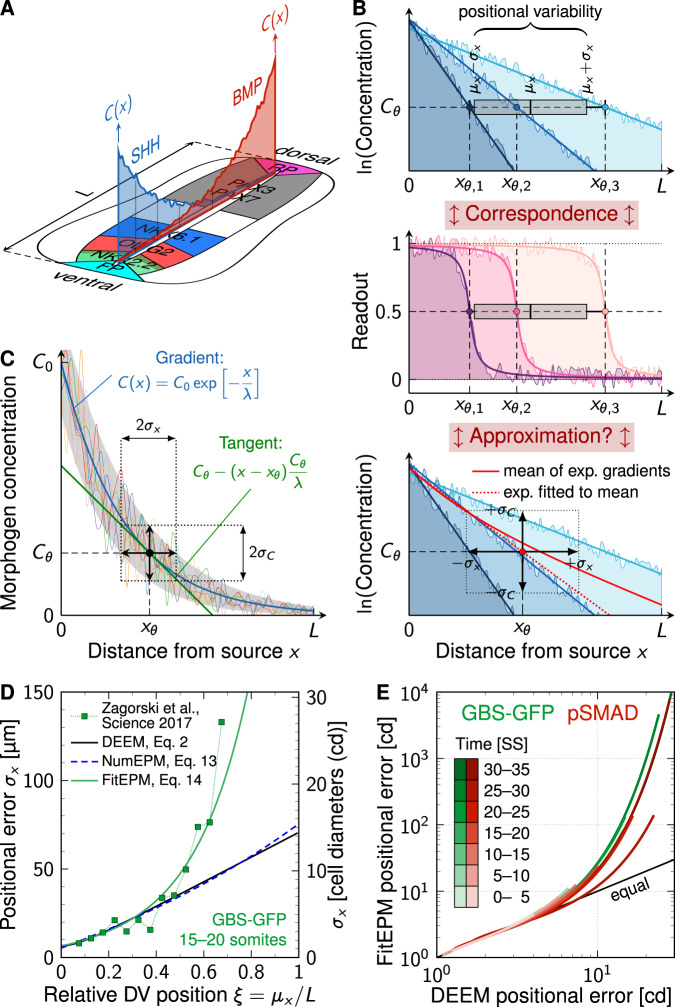
Estimation of positional error of morphogen gradients in the vertebrate neural tube. **A** Schematic of opposing noisy SHH and BMP gradients with resulting gene expression domains. The patterning domain is confined between the floor plate (FP) and the roof plate (RP). **B** Gradients from different embryos (top, different colors) differ in amplitude and decay length. As a result, the threshold concentration *C*_*θ*_ is reached at different readout positions *x*_*θ*,*i*_. The overall readout position, *μ*_*x*_, is given by the mean of the readout positions, *x*_*θ*,*i*_. The positional error, *σ*_*x*_, is the standard deviation of the readout positions *x*_*θ*,*i*_ (Eq. (2)). At the threshold concentration *C*_*θ*_, the readout (middle) of the gradient typically assumes its half-maximal value. If the precision of both gradient and readout is measured by the same error metric, there is a 1:1 correspondence between them. Noise in individual gradients can further widen the transition zone between two adjacent domains. With the FitEPM (bottom, Eq. (14)), individual exponential gradients are averaged (red solid line) and then lin-fitted with another exponential (red dotted line), to then apply linear error propagation to estimate the positional error. **C** In the linear error propagation method, the local tangent (green) to an averaged morphogen concentration gradient (blue) is used to translate the vertical gradient variability, *σ*_*C*_, into a horizontal positional error, *σ*_*x*_ (black). **D** Comparison of the previously reported positional error of GBS-GFP (green squares)^9^ with the results obtained with 10^4^ randomly drawn gradients that match the reported gradient summary statistics. The positional error is calculated either directly, Eq. (2) (DEEM, black solid line), or with the linear error propagation method, Eq. (3), using the derivative of either the mean gradient (Eq. (13), NumEPM, blue dashed line) or of an exponential function fitted to the mean gradient (Eq. (14), FitEPM, green solid line). See Methods for details. **E** When applied to sets of exponential gradients with mean and standard deviation of *C*_0_ and *λ* identical to those reported for GBS-GFP (green) and pSMAD (red), the FitEPM overestimates the positional error (Eq. (2), black) by orders of magnitude further away from the source. Source data are provided as a Source Data file.

During embryogenesis, tissues specialize, and cells in different subdomains take on different fates. Morphogen gradients can provide the positional information to define the boundaries of distinct progenitor domains (Fig. 1A). According to the French flag model, boundary positions are defined by threshold concentrations^1^. Molecular noise in morphogen production, transport, and decay affects the gradient shape such that the threshold concentration is reached at different readout positions in different embryos. How such variable gradients can encode precise spatial information, and to which degree they are accurate enough to pattern a developing tissue is an open question, despite intense studies^2–10^.

Given the challenges in visualizing morphogen gradients, detailed studies of gradient precision have only been carried out in few developmental systems. First and foremost, for the Bicoid gradient in the early *Drosophila* embryo^5^, the Decapentaplegic (Dpp) gradient in the *Drosophila* wing disc^6^, and for the Sonic hedgehog (SHH) and Bone morphogenetic protein (BMP) gradients in the mouse neural tube (NT)^9^. In all these systems, the morphogen gradients take on an exponential form^5,11–13^

$$C(x)={C}_{0}\exp \left[-\frac{x}{\lambda }\right]$$

with an amplitude *C*_0_ at the morphogen source at *x* = 0 and a decay length *λ*. In the French flag model, domain boundaries are positioned where the gradient concentration reaches a threshold concentration *C*_*θ*_ = *C*(*x*_*θ*_), i.e., at

1 $${x}_{\theta }=\lambda \ln \left[\frac{{C}_{0}}{{C}_{\theta }}\right].$$

The gradient amplitude *C*_0_ and length *λ* differ between embryos as a result of molecular noise in morphogen production, decay and diffusion^5,7,12,13^. Differences in gradient amplitude and decay length translate into differences in the readout position *x*_*θ*,*i*_ where the threshold concentration *C*_*θ*_ is reached in each embryo *i* (Fig. 1B, top). The overall readout position for the particular threshold concentration *C*_*θ*_ is the mean position *μ*_*x*_ = mean{*x*_*θ*,*i*_}. The positional error is given by the standard deviation (SD) of the readout positions in the different embryos,

2 $${\sigma }_{x}={{{{{{\mathrm{SD}}}}}}}\,\{{x}_{\theta ,i}\}.$$

Following this definition, the positional error of two centrally located NT progenitor domain boundaries, the dorsal NKX6.1 boundary and the ventral PAX3 boundary (Fig. 1A), was found to be about 1–3 cell diameters^9^. In parallel, also the gradient variability was measured, which has enabled the discussion of patterning precision in the NT. Due to challenges in measuring morphogen gradients directly^12,14^, GBS-GFP was used as transcriptional reporter of SHH signaling, and phosphorylated Smad1/3/5 (pSMAD) as a readout of BMP signaling. Close to the source, the positional error increased from a single cell diameter (4.9 μm^12^) to about three cell diameters over time. In the center of the NT, however, the positional error was reported to increase from 1 to 2 cell diameters in early stages to more than 30 cell diameters later on. Combined readout of the imprecise SHH and BMP gradients was proposed to yield the higher precision of the central progenitor boundaries^9^. However, even a combined readout fails after 15 somite stages (SS), i.e., after about 30 h of spinal cord development, and it remained unclear how the precise patterning of the central progenitor domains is achieved.

In this article, we revisit the problem of morphogen gradient readout and patterning precision. We show that the positional error of gradients in the NT has previously been overestimated, and demonstrate that a single morphogen gradient in the NT is sufficiently precise to define the NKX6.1 and PAX3 progenitor domain boundaries with the reported accuracy during the first day of NT development. We further show that technical limitations do not allow for precise gradient measurements in the center of the NT at later stages. We develop a theoretical framework and combine it with numerical computations to estimate gradient variability also at later stages of NT development. Based on the measured variabilities in morphogen production, turnover and diffusion, we infer the variability of morphogen gradients and thus their positional error as it results from molecular noise. The resulting positional accuracy is consistent with the observed precision of the readout boundaries in the mouse NT. The gradients are thus, in principle, sufficiently precise to yield the observed patterning precision. Furthermore, we show that the size of gene expression domains is independent of the activity and variability thereof when defined by the threshold-based readout of a single morphogen gradient. This results in a very robust mechanism to produce precise numbers of progenitor cells.

## Results

### Positional error of gradients was previously overestimated in the mouse NT

In principle, a wide range of error metrics could be defined, but in order to address the question whether a gradient is sufficiently precise to define a progenitor domain boundary, the variability of the gradient and of the progenitor domain boundary must be measured with the same metric. Zagorski et al.^9^ determined the positional error of the NKX6.1 and PAX3 domain boundaries by first determining their position in individual embryos, and by subsequently determining their standard deviation, *σ*_*x*_ (Eq. (2)). Going forward, we refer to Eq. (2) as the direct error estimation method (DEEM). For the gradient, on the other hand, a different, well-established^5–8,15,16^ approximation was used, given by the linear error propagation formula

3 $${\sigma }_{x}\approx {\left|\frac{\partial C}{\partial x}\right|}^{-1}{\sigma }_{C},$$

to estimate the positional error of gradients *C* from their slope, ∂*C*/∂*x*, and variance, ${\sigma }_{C}^{2}$, instead of using Eq. (2) directly with gradients. In this indirect way, vertical variability of the gradients (measured by their standard deviation, *σ*_*C*_ = SD[*C*(*x*_*θ*_)]) is translated into horizontal variability of the readout position (*σ*_*x*_ = SD[*x*_*θ*_]) by multiplication with the inverse slope of the mean gradient (Fig. 1B, C). We now test whether both approaches yield an equivalent result.

To determine the positional error with Eq. (3), an averaged inverse slope needs to be determined from the individual concentration or intensity profiles. Gregor et al.^5^ used numerical differentiation of the mean gradient to determine the derivative (personal communication), an approach that we refer to as numerical differentiation error propagation method (NumEPM for short). Bollenbach et al.^6^ and Zagorski et al. (^9^, personal communication) first fitted an exponential function to the mean gradient (Fig. 1B, bottom). Then, they exploited that for an exponential function,

$$\frac{\partial C}{\partial x}(x)=-\frac{C(x)}{\lambda },$$

such that ${\left|\partial C/\partial x\right|}^{-1}$ follows from the local value of the fitted exponential function and the fitted decay length *λ*. We use the acronym FitEPM to refer to this error propagation method.

If these approximations are appropriate, then both the NumEPM and the FitEPM should yield the same positional error as the DEEM. We can test this by applying all three methods to the same set of 10^4^ exponential gradients with different gradient amplitudes *C*_0,*i*_ and lengths *λ*_*i*_,

4 $${C}_{i}(x)={C}_{0,i}\exp \left[-\frac{x}{{\lambda }_{i}}\right].$$

To determine the positional error according to its mathematical definition (Eq. (2)), one needs to determine the positions, *x*_*θ*,*i*_ where each gradient reaches the threshold concentration *C*_*θ*_ (Fig. 1B, top). For the purely exponential gradients, there is only a single intersection and the readout position is thus unique. The readout position *μ*_*x*_ for a given concentration threshold *C*_*θ*_ is then given by the mean of all *x*_*θ*,*i*_, while the positional error at this readout position is given by the standard deviation (Eq. (2)). If this is repeated with threshold concentrations over the entire concentration range, the positional error along the domain is obtained (Fig. 1D, black line). While the NumEPM results in positional errors (Fig. 1D, blue dashed line) close to the DEEM (Fig. 1D, black line), the FitEPM yields much larger positional errors (Fig. 1D, green line), and strongly overestimates the positional error further away from the source (Fig. 1D, E). The difference between the NumEPM and the FitEPM arises from a subtle, but important difference. With the NumEPM, the slope of the mean gradient is obtained by numerical differentiation, whereas in the FitEPM, an exponential function is fitted to the mean gradient to estimate their slope. The arithmetic mean of (different) exponentials is, however, not exponential itself (Fig. 1B, bottom). There is no way to write

$$\frac{1}{n}\mathop{\sum }\limits_{i=1}^{n}{C}_{0,i}\exp \left[-\frac{x}{{\lambda }_{i}}\right]\mathop{=}\limits^{!}{C}_{0}\exp \left[-\frac{x}{\lambda }\right]$$

unless all decay lengths are equal, *λ* ≡ *λ*_*i*_. Further away from the source, an exponential function fitted to the mean of exponentials lies below that mean (Fig. 1B, bottom), when fitted without logarithmizing the data first. The inverse of this underestimated concentration then enters Eq. (3), which is why the positional error in^9^ is much higher than the error directly evaluated with Eq. (2) further away from the source.

The synthetic, purely exponential gradients (Eq. (4)) were drawn such that the statistical properties (mean and standard deviation) of the gradients are identical to those reported for GBS-GFP and pSMAD^9^ (see Methods). Even though the synthetic gradients do not include local noise or deviations from the exponential shape, the positional error obtained with the FitEPM with the synthetic data (Fig. 1D, green line) is very similar to that reported in^9^ for the real data (Fig. 1D, green squares). This suggests that it is mainly the computational method that generates the high apparent positional error rather than the additional deviations that are included in the measured, but not in the fitted gradients. We conclude that the accuracy of Eq. (3) hinges critically on the accuracy of the derivative, and that the FitEPM exaggerates the gradient positional error.

### Single gradients are sufficiently precise to define the central progenitor boundaries in the mouse neural tube

**Fig. 2:**
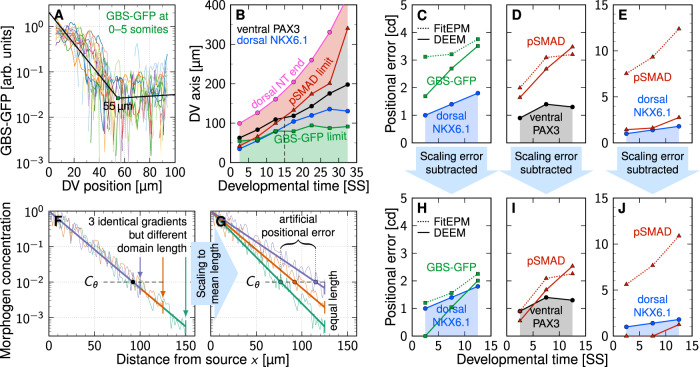
Single gradients are precise enough to define the central progenitor domain boundaries in the mouse neural tube. **A** The GBS-GFP and pSMAD gradients^9^ follow an exponential curve only close to the source and then transition to a noise bed, as revealed by fitting a kinked line (black) to the logarithmic intensity with the kink position as free parameter (green square). **B** Exponential limit as obtained from the kink position in the GBS-GFP (green) and pSMAD (red) intensity profiles over time. The dorsal NKX6.1 (blue) and ventral PAX3 (black) domain boundaries (extracted from^9^) lie within the exponential part of the gradients only during the first 15 somite stages (dashed line). Later, they fall in the noise-dominated central region (gray). DV position in (**A**,**B**) is measured from the ventral end. **C**,**D**,**E** Comparison of the reported positional error evolution of the dorsal NKX6.1 (**C**,**E**, blue) and ventral PAX3 (**D**, black) domain boundaries to the positional error of the GBS-GFP (**C**, green) and pSMAD (**D**,**E**, red) gradients along these domain boundaries. Gradient errors determined either with the FitEPM (Eq. (14), dotted lines) or the DEEM (Eq. (2), solid lines), in units of cell diameters (cd). The shaded region indicates the range into which the gradient positional errors (green, red) would need to fall for the gradients to be precise enough to directly explain the measured domain boundary precision. **F**,**G** Rescaling the domains to the mean domain length changes the decay length and introduces an artificial positional error. **H**,**I**,**J** After subtraction of the domain scaling error introduced in^9^, the gradients' positional errors (green, red) are sufficiently low to be consistent with the reported dorsal NKX6.1 (blue) and ventral PAX3 (black) boundary errors. Source data are provided as a Source Data file.

The measured GBS-GFP and pSMAD gradients differ from the fitted gradients that we considered in Fig. 1 not only in that they are noisy, but also in that they follow an exponential trend only within a certain proximity to the source, and then switch to a shallow noise bed (Fig. 2A). The dorsal NKX6.1 and ventral PAX3 boundaries lie within the exponential part of the gradients only for the first 15 SS (Fig. 2B). After that, the measured GBS-GFP and pSMAD gradients are too flat to convey positional information. This matches previous observations in^9^, where the positional accuracy of the dorsal NKX6.1 and ventral PAX3 could be explained with the opposing GBS-GFP and pSMAD gradients only during the first 15 SS, which corresponds to about 30 h of developmental time. We will now focus on the same first 15 SS.

Along the NKX6.1 and PAX3 boundaries, we find a positional error of <4 cell diameters for the gradient closer to the source, i.e., for GBS-GFP in case of the dorsal NKX6.1 boundary (Fig. 2C, green solid line) and for pSMAD in case of the ventral PAX3 boundary (Fig. 2D, red solid line). Similar results were reported also in^9^ (Fig. 2C, green dotted line, Fig. 2D, red dotted line). The FitEPM overestimates the positional error only when the readout position lies far from the source (Fig. 1D, E), as can be seen for the dorsal NKX6.1 boundary with the pSMAD gradient (Fig. 2E, red lines).

The reported positional error of the NKX6.1 and PAX3 boundaries is 1–2 cell diameters, which is slightly less than that of the gradients (Fig. 2C–E). The remaining difference between gradient and readout can be accounted to the preprocessing of the gradients in^9^, which artificially increases the positional error (Fig. 2F, G). The stage-matched gradients that we received from the authors of^9^ are binned in 5 SS, corresponding to 10 h of developmental time. Unlike the early *Drosophila* embryo analyzed in^5^, the mouse NT grows at about 10 μm per SS^9,12,17^ such that the domain length in each bin can be expected to differ by about 50 μm. The authors scaled the gradients in each bin to the average length. This rescaling of gradients changes the decay length *λ* and introduces an artificial positional error (Fig. 2F, G). Assuming uniform sampling of SS in each bin, the resulting artificial positional error is given by the standard deviation of a uniform distribution spanning 50 μm. As the domains were measured from the dorsal end in^9^, this is 50 μm$/(2\sqrt{3})\approx 14.4$ μm, or about three cell diameters, at the ventral end. Since the NT grows uniformly along the DV axis^17^, this implies a difference of 3(1 − *ξ*) cell diameters at position *ξ* in the domain (with *ξ* = *x*/*L* = 0 at the ventral end). After subtraction of this artificial error from the inferred positional error, we obtain values that are very close to what has been reported for PAX3 and NKX6.1, or even lower (Fig. 2H–J).

We conclude that the SHH gradient is sufficiently precise to define the dorsal NKX6.1 boundary, which together with NKX6.2 and DBX1 represses *Pax3*^18,19^. The BMP gradient appears sufficiently precise to define either boundary in the center of the NT.

### Technical limits to gradient detection

**Fig. 3:**
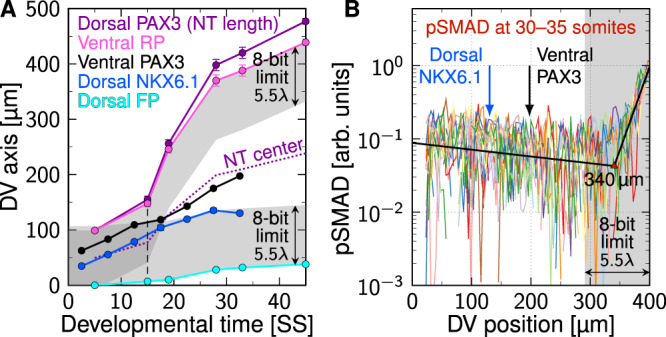
Technical limits to gradient detection. **A** NT length (purple) and position of domain limits over developmental time. At later stages, the dorsal NKX6.1 (blue) and ventral PAX3 (black) domain boundaries lie at the edge or outside the 5.5*λ* = 107 μm detection limit of 8-bit microscopy (shadowed). The pink and cyan lines mark the limit of the BMP-secreting roof plate (RP) and SHH-secreting floor plate (FP), respectively. The FP and RP lengths were assumed to be equal. The analyzed first 15 somite stages (SS, dashed) coincide with the time window over which 8-bit detection suffices to cover the entire NT. Data are presented as mean values ± SEM from *n* = 18, 42, 31, 6, 24, 13, 13 measurements for dorsal NKX6.1, *n* = 22, 33, 42, 15, 32, 38, 28 for ventral PAX3, *n* = 123 for dorsal PAX3 and FP/RP, reproduced from^9,17^. **B** The measured GBS-GFP and pSMAD gradients^9^ contain an approximately constant noise bed that dominates the signal from within the 8-bit detection limit (shadowed) onward. The dorsal NKX6.1 and ventral PAX3 domain boundaries (arrows) lie far out in the noise bed at later stages. Source data are provided as a Source Data file.

The analysis was restricted to the first 15 SS (≈30 h) of NT development because the central progenitor domain boundaries lie in the noise bed of the measured GBS-GFP and pSMAD gradients afterwards (Fig. 2B). It is not known whether the shallow part of the GBS-GFP and pSMAD gradients (Fig. 2A, B) reflects similar behavior of the SHH and BMP gradients, and if so, whether it reflects the actual gradient shape or technical limitations. As quantitative imaging of exponential morphogen gradients is challenging^12,14^, much speaks in favor of technical limitations. While cells have evolved multi-threshold and adaptive readouts^12,20^, microscopes are more limited. With 8-bit images, as recommended in a subsequent protocol paper for NT gradient measurements^21^, at most a 256-fold signal range can be detected, corresponding to an exponential decay over the range of about 5.5 *λ*, which is 107 μm ≈ 22 cell diameters in the mouse NT (Fig. 3A, shaded region). Any 8-bit visualization of exponential gradients will necessarily miss the exponential character beyond that distance. In practice, the usable range will even be shorter, if technical noise occupies a few percent of the 8-bit channel. As the same settings were used in all measurements, the decline of the GBS-GFP and pSMAD gradient amplitudes over developmental time^9^ further restricts the detection range at later times such that also the dorsal NKX6.1 boundary will lie outside the GBS-GFP detection range. Indeed, the gradient noise is equal everywhere in the noise bed, including the region where the detection of an exponential gradient with 8-bit imaging is certainly impossible (Fig. 3B). While 16-bit imaging would be possible, at least the SHH reporter GBS-GFP reporter poses further limits. With its eight concatemerized fragments of a *FoxA2* enhancer that contains a GLI binding site^22^, GBS-GFP necessarily follows the SHH response of *FoxA2*. *FoxA2* requires high SHH concentrations, and its expression is thus restricted to the ventral-most SHH-secreting floor plate and the adjacent p3 domain^23^. While GBS-GFP extends beyond the p3 domain, presumably because the fragments lack additional negative regulatory elements of the full *FoxA2* enhancer, GBS-GFP will have the same strong dependency on GLIA input as *FoxA2*. Other SHH-responsive genes that do not depend on GLIA input are well known to be expressed at much lower SHH concentrations than *FoxA2*. Accordingly, the transition to the noise bed could either indicate the limits of 8-bit imaging or the response limits of GBS-GFP. In both cases, technical limitations preclude the detection of exponential gradient profiles in the center of the NT.

But are long-range exponential gradients plausible, and could cells detect such low concentrations? In the developing vertebrate NT, positional information is provided by opposing SHH and BMP gradients^24^ (Fig. 1A). If each morphogen patterns only one half of the domain, the morphogen concentration in the center of the final domain will be about 10^4^-fold lower than at the source. In the bacterial chemotaxis response, adaptation allows cells to sense concentration gradients spanning at least five orders of magnitude, and cooperativity in receptor clusters enables a high gain such that the occupancy of one or two receptors can be sensed^25^. Whether similar effects are at work also in the NT is unclear, but adaptation in the SHH responsiveness has been noted^12,20^, and the PTCH1 receptor organizes as dimer of dimers, and each dimer binds one SHH^26^. Accordingly, it is, in principle, possible that morphogens can be sensed by cells over a 10^5^-fold concentration range (11.5 *λ*), which corresponds to about 220 μm in the mouse NT—enough to cover the entire NT domain with opposing gradients (Fig. 3A). But even if the gradients can be sensed over 11.5 *λ*, how precise would the conveyed positional information be? We will now take a computational approach to estimate the gradient variability based on available data.

### Estimation of positional error from statistical gradient properties

Close to the source, the measured gradients can be approximated well by exponential functions^9^, and the positional error of the fitted exponentials is similar to that of the raw gradients (Fig. 1). In the following, we show how the positional error can be calculated from the summary statistics of the exponential gradients rather than by evaluating the standard deviation of individual gradients. This then allows to predict the positional error of the gradients at a distance from the source based on the observed variability closer to the source, assuming that the exponential gradient shape is maintained. With this formalism we can then infer the maximal gradient variability that would be consistent with the observed readout precision in the mouse NT.

**Fig. 4:**
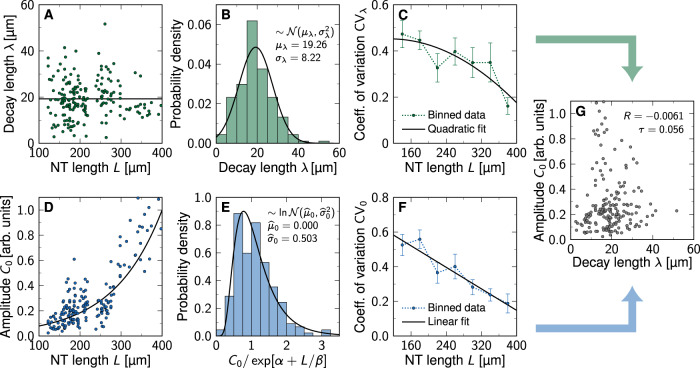
Statistical properties of the SHH gradient in the developing mouse neural tube. **A**,**B** *λ* is constant over developmental time, consistent with a truncated normal distribution. **C** Binning the data into 40 μm bins reveals that the relative variability in the data drops over time for SHH. **D** *C*_0_ increases as the neural tube expands. The solid line shows an exponential fit $\exp [\alpha +L/\beta ]$. **E** Relative to the growing mean, the variability in the amplitude data is consistent with a log-normal distribution. **F** Also the relative amplitude variability of SHH declines over time. Data in (**C**,**F**) are presented as mean values ± bootstrapped SEM (samples per bin: *n* = 31, 63, 22, 36, 11, 12, 6). **G** SHH gradient length and amplitude are uncorrelated (Pearson’s correlation coefficient *R* ≈ 0, Kendall’s *τ* ≈ 0). Data points reproduced from^12^. Source data are provided as a Source Data file.

The reported *λ* values for SHH in the mouse NT (Fig. 4A) are consistent with a (truncated) normal distribution (Fig. 4B). We therefore consider *λ* as a Gaussian random variable with mean *μ*_*λ*_ and standard deviation *σ*_*λ*_. While the mean value remains roughly constant at about 20 μm over developmental time (Fig. 4A), the deviation from the mean, as measured by the coefficient of variation CV_*λ*_ = *σ*_*λ*_/*μ*_*λ*_, has been reported to drop as the NT grows (Fig. 4C). At a given point in time (i.e., at a given size of the NT), the available data (Fig. 4D) suggests that the amplitude *C*_0_ is log-normally distributed (Fig. 4E). While the mean amplitude *μ*_0_ increases over developmental time (Fig. 4D), the deviation from the mean, as measured by the coefficient of variation CV_0_ = *σ*_0_/*μ*_0_, reportedly drops as the NT grows (Fig. 4F). The inferred statistical parameters are summarized in Table 1. Similar data for the opposing BMP gradient is not available, but once it so becomes, our formalism is likely to apply analogously to BMP, as it also diffuses into the NT.

**Table 1:** Fitted statistical gradient parameters for the mouse neural tube.

Gene/signal	Mean length *μ*_*λ*_	Coeff. of variation CV_*λ*_ = *σ*_*λ*_/*μ*_*λ*_	Coeff. of variation CV_0_ = *σ*_0_/*μ*_0_	Source
SHH	19.26 μm	$-{\left(\frac{L}{547.9\,\mu {{{{{{{\rm{m}}}}}}}}}\right)}^{2}+\frac{L}{1341\,\mu {{{{{{{\rm{m}}}}}}}}}+0.410$	$-\frac{L}{644.1\,\mu {{{{{{{\rm{m}}}}}}}}}+0.769$	^12^
GBS-GFP	19.43 μm	$-{\left(\frac{L}{819.6\,\mu {{{{{{{\rm{m}}}}}}}}}\right)}^{2}+\frac{L}{861.0\,\mu {{{{{{{\rm{m}}}}}}}}}+0.128$	${\left(\frac{L}{790.8\,\mu {{{{{{{\rm{m}}}}}}}}}\right)}^{2}-\frac{L}{3437\,\mu {{{{{{{\rm{m}}}}}}}}}+0.313$	^9^
pSMAD	22.67 μm	$-{\left(\frac{L}{820.3\,\mu {{{{{{{\rm{m}}}}}}}}}\right)}^{2}+\frac{L}{800.0\,\mu {{{{{{{\rm{m}}}}}}}}}+0.072$	${\left(\frac{L}{573.1\,\mu {{{{{{{\rm{m}}}}}}}}}\right)}^{2}-\frac{L}{722.2\,\mu {{{{{{{\rm{m}}}}}}}}}+0.401$	^9^

Since morphogen concentrations are measurable only in arbitrary units (Fig. 4D) and since exponentials remain exponential independent of the chosen absolute scale, we can normalize the gradients by an arbitrary reference concentration without loss of generality. In the absence of precise knowledge about the readout threshold *C*_*θ*_, we choose the concentration scale such that *C*_*θ*_ = 1 in the following, which simplifies the notation. Our results retain their validity for general *C*_*θ*_. Hence, we assume that also the ratio *C*_0_/*C*_*θ*_ follows a log-normal distribution:

$$\frac{{C}_{0}}{{C}_{\theta }} \sim {{{{{{\mathrm{Logn}}}}}}}\,({\widehat{\mu }}_{0},{\widehat{\sigma }}_{0}^{2})\quad \iff \quad \ln \left[\frac{{C}_{0}}{{C}_{\theta }}\right] \sim {{{{{{{\mathcal{N}}}}}}}}({\widehat{\mu }}_{0},{\widehat{\sigma }}_{0}^{2}).$$

Here, ${\widehat{\mu }}_{0}$ and ${\widehat{\sigma }}_{0}$ are the mean and standard deviation of the Gaussian random variable $\ln [{C}_{0}/{C}_{\theta }]$. We can use the properties of log-normal distributions^27^ to express ${\widehat{\mu }}_{0}$ and ${\widehat{\sigma }}_{0}$ in terms of the mean *μ*_0_ and standard deviation *σ*_0_ of *C*_0_/*C*_*θ*_:

5 $${\widehat{\mu }}_{0}=\ln {\mu }_{0}-\frac{{\widehat{\sigma }}_{0}^{2}}{2}\quad \,{{\mbox{and}}}\,\quad {\widehat{\sigma }}_{0}^{2}=\ln \left[1+\frac{{\sigma }_{0}^{2}}{{\mu }_{0}^{2}}\right]$$

where

$${\mu }_{0}={\mathbb{E}}\left[\frac{{C}_{0}}{{C}_{\theta }}\right]\quad \,{{\mbox{and}}}\,\quad {\sigma }_{0}^{2}={{{{{{\mathrm{Var}}}}}}}\,\left[\frac{{C}_{0}}{{C}_{\theta }}\right].$$

To estimate how domain boundaries behave under variability in the morphogen gradient, we seek to express the expected boundary position ${\mu }_{x}={\mathbb{E}}\left[{x}_{\theta }\right]$ and its standard deviation ${\sigma }_{x}={{{{{{\mathrm{SD}}}}}}}\,\left[{x}_{\theta }\right]$ as functions of the four gradient parameters *μ*_*λ*_, *σ*_*λ*_, *μ*_0_, *σ*_0_. The data for the SHH gradient in the mouse NT suggests that the gradient’s decay length and amplitude are uncorrelated (Pearson’s *R* = −0.0061, Kendall’s *τ* = 0.056, Fig. 4G). This allows us to exploit the multiplicative properties of two independent random variables *X* and *Y*,

6 $${\mathbb{E}}\left[XY\right]={\mathbb{E}}\left[X\right]{\mathbb{E}}\left[Y\right]$$

and

7 $${{{{{{\mathrm{Var}}}}}}}\,\left[XY\right]={{{{{{\mathrm{Var}}}}}}}\,\left[X\right]{{{{{{\mathrm{Var}}}}}}}\,\left[Y\right]+{{{{{{\mathrm{Var}}}}}}}\,\left[X\right]{\mathbb{E}}{\left[Y\right]}^{2}+{{{{{{\mathrm{Var}}}}}}}\,\left[Y\right]{\mathbb{E}}{\left[X\right]}^{2}.$$

Putting Eqs. (1), (5) and (6) together, the mean boundary position is given by

8 $${\mu }_{x}={\mu }_{\lambda }{\widehat{\mu }}_{0}={\mu }_{\lambda }\ln \left[\frac{{\mu }_{0}}{\sqrt{1+{\sigma }_{0}^{2}/{\mu }_{0}^{2}}}\right].$$

If there is no variability in *C*_0_/*C*_*θ*_ (i.e., ${\sigma }_{0}={\widehat{\sigma }}_{0}=0$), then Eq. (8) reduces to the deterministic case, Eq. (1).

The squared positional error follows from combining Eqs. (1), (5) and (7):

9 $${\sigma }_{x}^{2} 	={\sigma }_{\lambda }^{2}{\widehat{\sigma }}_{0}^{2}+{\sigma }_{\lambda }^{2}{\widehat{\mu }}_{0}^{2}+{\widehat{\sigma }}_{0}^{2}{\mu }_{\lambda }^{2}\\ 	=\left({\mu }_{\lambda }^{2}+{\sigma }_{\lambda }^{2}\right)\ln \left[1+\frac{{\sigma }_{0}^{2}}{{\mu }_{0}^{2}}\right]+{\left({\sigma }_{\lambda }\ln \left[\frac{{\mu }_{0}}{\sqrt{1+{\sigma }_{0}^{2}/{\mu }_{0}^{2}}}\right]\right)}^{2}.$$

**Fig. 5:**
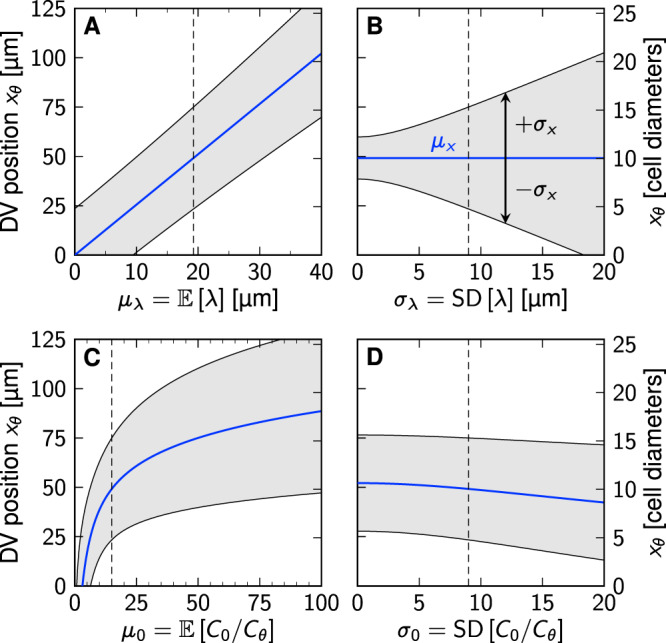
Effect of concentration gradient parameters on domain boundary position and positional error. Equations (8) (blue) and (9) (gray) are plotted as a function of the mean gradient length (**A**), its standard deviation (**B**), the mean relative amplitude (**C**), and its standard deviation (**D**). Each panel shows the variation of one parameter, with the other three fixed at measured early SHH values in mouse: *μ*_*λ*_ = 19.26 μm, *σ*_*λ*_ = 9 μm, *μ*_0_ = 15, *σ*_0_ = 9 (indicated by dashed lines).

Notably, *σ*_0_ enters the position and positional error of a domain boundary only through the coefficient of variation CV_0_ = *σ*_0_/*μ*_0_. Equations (8) and (9) provide direct insight into how the statistical distributions of the gradient length and amplitude impact on the location and variability of the readout position (Fig. 5). The larger *σ*_0_, the smaller *μ*_*x*_ (Fig. 5D), i.e., the further the domain boundary shifts upward the concentration gradient, toward the morphogen source. Variability in the decay length *λ*, on the other hand, leaves the mean boundary position unaffected (Fig. 5B), as Eq. (8) is independent of *σ*_*λ*_. A larger mean gradient length or amplitude shifts the boundary downhill, away from the source (Fig. 5A, C). The positional error, on the other hand, depends on both gradient parameters and their scatter in a complicated nonlinear fashion that can even be non-monotonic.

We can further substitute Eq. (8) into Eq. (9) to obtain the positional error as an explicit function of the boundary position:

10 $${\sigma }_{x}^{2}={\mu }_{\lambda }^{2}\left(1+{{{{{{{{\rm{CV}}}}}}}}}_{\lambda }^{2}\right)\ln \left[1+{{{{{{{{\rm{CV}}}}}}}}}_{0}^{2}\right]+{{{{{{{{\rm{CV}}}}}}}}}_{\lambda }^{2}{\mu }_{x}^{2}.$$

Equation (10) is by construction identical to the direct way of computing the positional error via Eq. (2) (DEEM) from infinitely many gradients.

The positional error as a function of its readout position *μ*_*x*_, as given by Eq. (10), is independent of the mean gradient amplitude *μ*_0_. Precise knowledge of the change of *μ*_0_ over time (or as a function of *L*) is therefore not required to predict the positional accuracy in a noisy morphogen gradient. All that is needed is the variation of the amplitude relative to its mean, CV_0_. This has several beneficial consequences. A practical one is that no absolute measurement of the gradient amplitude is needed from experiments—relative values are sufficient to quantify positional accuracy. Another convenience is that the exact functional relationship used to fit or model the change of *μ*_0_ over time or length, be it exponential as in Fig. 4D, linear as in^12^, or any other form, has no effect on the positional accuracy, as long as CV_0_ is given. Third, the fact that the absolute scale of the gradient amplitude is irrelevant implies that positional accuracy is unaffected by temporal changes in morphogen abundance, as long as CV_0_ remains sufficiently low.

### Precision of gradient readout boundaries in the NT

**Fig. 6:**
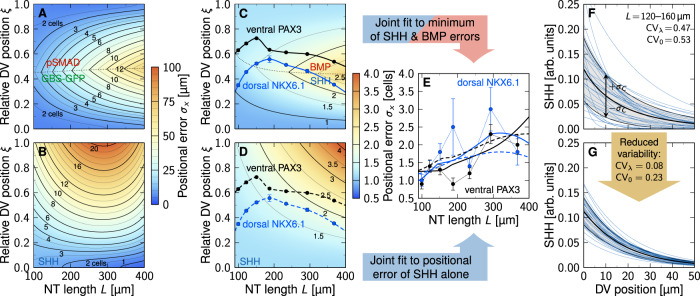
Gradient and readout imprecision in the developing neural tube. **A**,**B** Contour plots of the positional error *σ*_*x*_ (Eq. (10)) using reported GBS-GFP and pSMAD (**A**) and SHH (**B**) parameters from Table 1, as a function of the relative position along the DV axis, *ξ* = *μ*_*x*_/*L*, and the NT length, *L*. With the reported gradient variability, the positional error of the opposing SHH and BMP gradients is in the order of several cell diameters. **C**,**D** Dorsal NKX6.1 (blue) and ventral PAX3 (black) domain boundary positions, overlaid on the gradient precision contours obtained by fitting Eq. (10) to the measured positional errors of the boundaries (**E**). **A** and **C** Show the case where the positional error is the minimum of the two opposing gradients; the dotted line divides the NT into two parts in which either SHH or BMP provide higher accuracy. **B** and **D** Use only the SHH gradient. Black contours in (**A**–**D**) trace cell diameter isolines as labeled. **E** Reported (symbols) and predicted boundary precision if the domain boundaries are either set by SHH and BMP (solid lines, **C**) or by SHH alone (dashed lines, **D**). Domain boundary data in (**C**–**E**) are presented as mean values ± SEM calculated as detailed in Methods from *n* = 18, 42, 31, 6, 24, 13, 13 measurements for dorsal NKX6.1 and *n* = 22, 33, 42, 15, 32, 38, 28 for ventral PAX3, reproduced from^9^. **F**,**G** The exponential SHH gradients with {*λ*_*i*_, *C*_0,*i*_} as reported in^9^ are widely scattered in early NT development. The SHH gradients that match the measured positional error of the readouts (**C**,**E**) are still variable, but do not contain outliers. The black line represents the mean gradient, shaded areas show standard deviations *σ*_*C*_. Source data are provided as a Source Data file.

With Eq. (10), the precision of a domain boundary is fully determined by its relative location in the patterning domain, *ξ* = *μ*_*x*_/*L*, the domain length *L*, the mean decay length *μ*_*λ*_, and the coefficients of variation of the gradient length and amplitude, CV_*λ*_ and CV_0_. As estimates for the latter three are known from measurements (Fig. 4, Table 1), we can predict the boundary precision in the growing NT at any point in development, anywhere in the patterning domain. For the reported gradient variabilities, the positional error in the center of the NT becomes as high as 15 cell diameters over time (Fig. 6A, B, black contours). The reported precision of the PAX3 and NKX6.1 domain boundaries (1–3 cell diameters) is more likely to be correct than that of the gradients as the steep boundaries and the concomitant change in the fluorescent signal are much easier to detect. We used numerical optimization to determine the variability at which the positional accuracy of the SSH and BMP gradients together, or one of them alone, would be consistent with the reported positional accuracy of the NKX6.1 and PAX3 domain boundaries. Minimizing the difference between predicted (Eq. (10), color gradient & contour lines in Fig. 6C, D) and measured positional errors (data points in Fig. 6E) along the boundary positions (black & blue data in Fig. 6C, D) yielded optimal values for the gradient variability, CV_*λ*_ and CV_0_. Assuming that the boundaries are always defined by the more precise gradient, we can reproduce the boundary precision with CV_*λ*_ = 0.08 ± 0.04, CV_0_ = 0.23 ± 0.33 for SHH and CV_*λ*_ = 0.06 ± 0.04, CV_0_ = 0.26 ± 0.16, 95% C.I. for BMP (Fig. 6C, E). Remarkably, fitting the reported boundary precision to the SHH gradient alone yields similar CV values, CV_*λ*_ = 0.05 ± 0.03, CV_0_ = 0.30 ± 0.20, 95% C.I. (Fig. 6D, E), challenging the previously proposed idea that opposing gradients serve to increase positional accuracy in the NT^9^.

The inferred CV values for the gradients were used to plot the contours in Fig. 6C, D, and they lie near the lowest measured SHH gradient variability (Fig. 4C, F). Visual inspection shows that the reported variability corresponds to gradient profiles with some very short and some very long gradients that are difficult to reconcile with a successful patterning process (Fig. 6F). The variability inferred by us, while still resulting in variable gradients, does not result in such outliers (Fig. 6G). This raises the question whether the reported outliers reflect biological variation or technical problems in reliably measuring the morphogen gradients. Or differently put, how accurate are the reported gradient variabilities?

### Technical limitations in measuring morphogen gradient variability

**Fig. 7:**
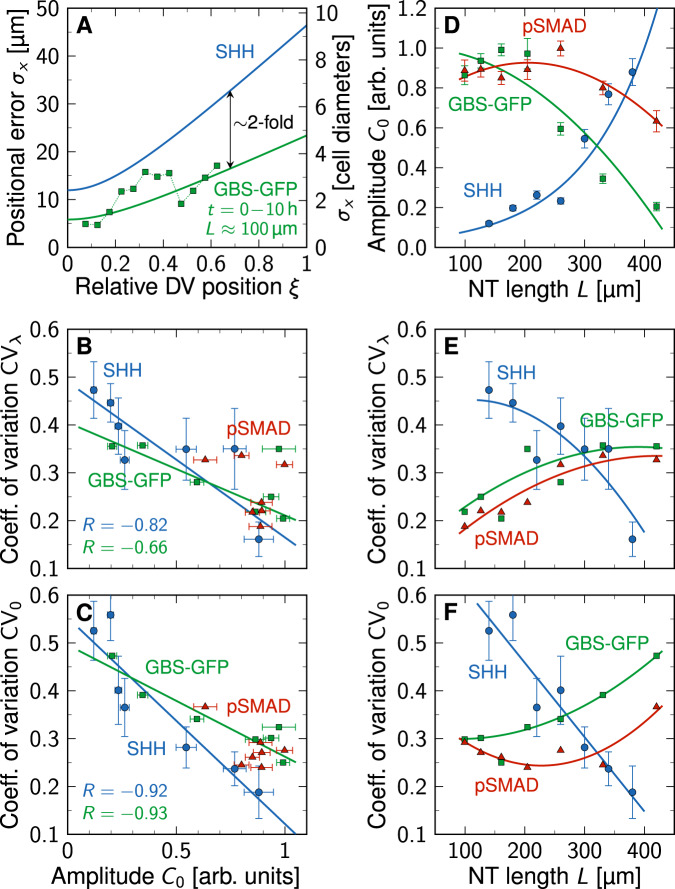
Technical limitations in measuring morphogen gradients. **A** With the reported variability, the SHH (blue) gradients would be about twice as imprecise as its readout GBS-GFP (green). The first developmental timepoint (0–10 h) of the GBS-GFP data (symbols) was reproduced from^9^; solid lines represent Eq. (10) with GBS-GFP and SHH parameters inferred from^9,12^ (Table 1). **B**,**C** Gradient variability is anti-correlated with the amplitude (Pearson correlation coefficient *R* ≪ 0), hinting at a potential technical limitation in the fluorescence intensity measurements. **D** The reported amplitudes of the SHH gradient increases, while the amplitudes of the GBS-GFP and pSMAD gradients decrease as the NT expands. **E**,**F** The reported gradient variabilities show the opposite trend. Solid lines are polynomial least-squares fits as listed in Table 1. Data in (**B**–**F**) are presented as mean values ± SEM from *n* = 31, 63, 22, 36, 11, 12, 6 (SHH, blue), *n* = 30, 62, 69, 17, 42, 32, 21 (GBS-GFP, green), *n* = 24, 40, 46, 19, 55, 33, 19 (pSMAD, red) measurements per bin, reproduced from^9,12^. Source data are provided as a Source Data file.

According to the reported gradient properties (Table 1), the SHH gradient is only about half as precise as the GBS-GFP gradient (Fig. 7A), even though GBS-GFP is a direct SHH reporter^22^, and the data pre-processing artificially increased its gradient length variability (Fig. 2). We emphasize that this difference is observed already at the earliest developmental timepoint, long before adaptation results in the down-regulation of the SHH-dependent response^20^. The difference may reflect temporal averaging for the GBS-GFP reporter (as would then also be expected for the other gradient readouts), and/or a higher technical variation for the SHH antibody staining than for the GBS-GFP reporter. The antibody staining for SHH in the NT patterning domain is very weak compared to that in the notochord, making gradient measurements challenging.

In support of technical limitations in determining gradient variability, the coefficients of variation are strongly negatively correlated with the intensity of the signal for SHH and GBS-GFP (Fig. 7B, C), even though the gradient amplitude increases for SHH and decreases for GBS-GFP and pSMAD over developmental time due to adaptation^20,28^ (Fig. 7D), while the coefficients of variation show the opposite trend (Fig. 7E, F). This suggests that technical limitations at low concentrations artificially increase the reported variability, precluding an accurate measurement of the true gradient variability. We therefore turned to simulations to infer the expected variability based on the reported variability of morphogen production, degradation and transport rates.

### Gradient variability as a result of molecular noise

**Fig. 8:**
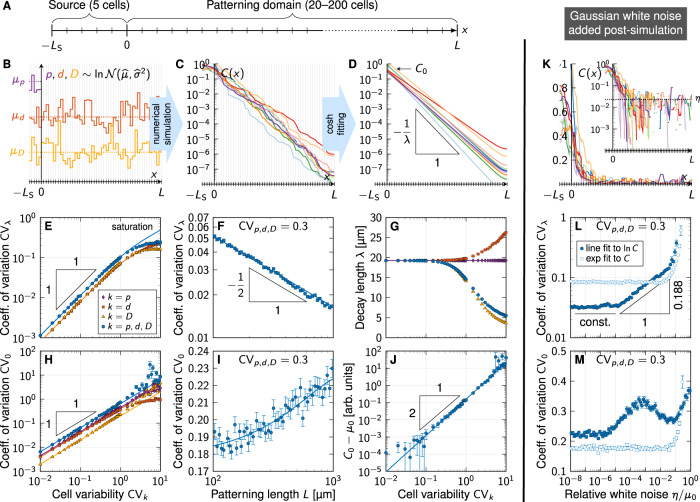
Numerical model predicts gradient variability from molecular noise. **A** Schematic of the simulated 1D domain. **B** Kinetic parameters *k* = *p*, *d*, *D* (purple, red, orange) were drawn randomly and independently for each cell from log-normal distributions with specified mean *μ*_*k*_ and coefficient of variation CV_*k*_. **C** Solving the reaction-diffusion equation repeatedly yields noisy morphogen gradients, from which the decay length *λ* and amplitude *C*_0_ can be extracted by fitting hyperbolic cosines in the patterning domain (0 ≤ *x* ≤ *L*, **D**). **E** The resulting variability in *λ* grows linearly with the variability in the kinetic parameters as long as CV_*k*_ ≲ 1, and saturates as CV_*k*_ increases further. **F** The length of the domain over which noisy gradients are fitted affects the variability of *λ* according to the law of large numbers, ${{{{{{{{\rm{CV}}}}}}}}}_{\lambda } \sim 1/\sqrt{L}$. **G** Increasing molecular noise leads to a bias in the resulting fitted *λ*. **H** The amplitude variability also increases linearly with CV_*k*_, but does not saturate if all three parameters have a variability exceeding one. **I** The variability of the fitted amplitude moderately grows with increasing patterning domain length. **J** Noisy parameters also induce an overestimation of the amplitude deduced from fitting, proportional to ${{{{{{{{\rm{CV}}}}}}}}}_{k}^{2}$. **K** Gaussian white noise $\sim {{{{{{{\mathcal{N}}}}}}}}(0,{\eta }^{2})$ added to the solution in all cells limits the range over which a line can be fitted to $\ln C$. **L** Lin-fitting (open circles) always (also at *η* = 0) leads to increased decay length variability, in particular with white noise stronger than one percent of the amplitude. Log-fitting (closed circles) is insusceptible to white noise as long as *η* ≲ 10^−5^*μ*_0_, and increases variability according to a power law with stronger white noise. If *η* exceeds a few percent of the amplitude, both fitting methods yield increased gradient length variability. **M** Amplitude variability is constant with lin-fitting for *η* ≲ 0.1*μ*_0_, whereas log-fitting yields larger CV_0_ values. *L* = 50 cells in all panels except (**F**,**I**). Data in (**E**–**J**,**L**,**M**) are presented as mean values ± SEM from *n* = 10^3^ independent simulations for each data point. Source data are provided as a Source Data file.

In a cellular tissue, the morphogen production, degradation, and transport rates vary from cell to cell. This variability ultimately generates the variability in the steady-state morphogen gradient profiles. We can estimate this variability by simulating a simple reaction-diffusion model on a continuous 1D domain where these parameter values differ randomly from segment to segment (Fig. 8A). To describe the steady-state morphogen profiles, we solve the steady-state reaction-diffusion equation

$$pH(-x)-dC(x)=-D\frac{{\partial }^{2}C}{\partial {x}^{2}}(x),\quad x\in [-{L}_{{{{{{{{\rm{S}}}}}}}}},L]$$

on a one-dimensional domain that was split into two subdomains, a morphogen source (−*L*_S_ ≤ *x* ≤ 0) and a patterning region (0 ≤ *x* ≤ *L*). The Heaviside step function *H* ensures that morphogen is produced at rate *p* only in the source, whereas it degrades at a linear rate *d* everywhere. Morphogen transport is driven by Fickian diffusion with diffusivity *D*. With zero-flux boundary conditions

$$\frac{\partial C}{\partial x}(-{L}_{{{{{{{{\rm{S}}}}}}}}})=0=\frac{\partial C}{\partial x}(L),$$

the deterministic solution is given by a concentration profile that follows hyperbolic cosines:

$$C(x)=\,	 \frac{p}{d}\left(H(-x)\left(1-\cosh \left[\frac{x}{\lambda }\right]\right)\right.\\ 	\left.+\frac{\sinh \left[{L}_{{{{{{{{\rm{S}}}}}}}}}/\lambda \right]}{\sinh \left[({L}_{{{{{{{{\rm{S}}}}}}}}}+L)/\lambda \right]}\cosh \left[\frac{L-x}{\lambda }\right]\right).$$

The decay length $\lambda =\sqrt{D/d}$ depends on the morphogen diffusivity *D* and the turnover rate *d*. The $\cosh$ is nearly exponential in the patterning domain except for a small deviation in the far end *x* ≈ *L* due to the zero-flux boundary. In the infinite size limit *L* → *∞*, a pure exponential emerges for *x* ≥ 0:

$$C(x)={C}_{0}\exp \left[-\frac{x}{\lambda }\right]\quad \,{{\mbox{with}}}\,\quad {C}_{0}=\frac{p}{2d}\left(1-\exp \left[-\frac{2{L}_{{{{{{{{\rm{S}}}}}}}}}}{\lambda }\right]\right).$$

In our simulations, we divided both subdomains into cells of length 4.9 μm, the average cell diameter in the mouse NT^12^ (Fig. 8A), and assigned each cell its own value of the three kinetic parameters *k* = *p*, *d*, *D*, drawn independently from log-normal distributions with prescribed means *μ*_*k*_ and coefficients of variation CV_*k*_ (Fig. 8B). Repeating the simulations many times for various CV_*k*_ values yielded independent noisy gradients spanning many orders of magnitude (Fig. 8C), from which we extracted *λ* and *C*_0_ by log-fitting hyperbolic cosines (Fig. 8D). We set ${\mu }_{p}={\mu }_{d}={\mu }_{D}/{\mu }_{\lambda }^{2}$ such that the deterministic decay length is the reported one of SHH, *μ*_*λ*_ = 19.26 μm^12^. The exact values of the parameters do not affect the steady-state result as long as the relationship is maintained; we chose as mean parameter *μ*_*D*_ = 0.033 μm^2^/s as measured for Hedgehog (Hh) in the *Drosophila* wing disc^29^ and fixed *μ*_*p*_, *μ*_*d*_ accordingly. The default setup consisted of 5 cells in the source, and 50 cells in the patterning domain.

This procedure yields the two gradient parameters and their variability as they result from molecular noise and NT expansion. We observe a linear increase of CV_*λ*_ as the cell variabilities CV_*d*,*D*_ are increased individually (keeping all others at zero), or all of them together, up to CV_*d*,*D*_ ≈ 1 (Fig. 8E). The production rate *p* affects only *C*_0_, not *λ*. This relationship can be understood theoretically. Since any product of powers of log-normal random variables is itself log-normal, so is $\lambda =\sqrt{D/d}$:

$$d \sim {{{{{{\mathrm{Logn}}}}}}}\,({\widehat{\mu }}_{d},{\widehat{\sigma }}_{d}^{2}),\,D \sim {{{{{{\mathrm{Logn}}}}}}}\,({\widehat{\mu }}_{D},{\widehat{\sigma }}_{D}^{2})\,\Rightarrow \,\lambda \sim {{{{{{\mathrm{Logn}}}}}}}\,({\widehat{\mu }}_{\lambda },{\widehat{\sigma }}_{\lambda }^{2})$$

with

$${\widehat{\mu }}_{\lambda }=\frac{{\widehat{\mu }}_{D}-{\widehat{\mu }}_{d}}{2},\qquad {\widehat{\sigma }}_{\lambda }^{2}=\frac{{\widehat{\sigma }}_{D}^{2}+{\widehat{\sigma }}_{d}^{2}}{4}$$

and

$${\widehat{\mu }}_{k}=\ln \left[\frac{{\mu }_{k}}{\sqrt{1+{{{{{{{{\rm{CV}}}}}}}}}_{k}^{2}}}\right],\quad {\widehat{\sigma }}_{k}^{2}=\ln \left[1+{{{{{{{{\rm{CV}}}}}}}}}_{k}^{2}\right],\quad k=d,D.$$

Using the expectation value and variance of log-normal distributions^27^,

$${\mu }_{\lambda }=\exp \left[{\widehat{\mu }}_{\lambda }+\frac{{\widehat{\sigma }}_{\lambda }^{2}}{2}\right],\quad {\sigma }_{\lambda }^{2}={\mu }_{\lambda }^{2}\left(\exp \left[{\widehat{\sigma }}_{\lambda }^{2}\right]-1\right),$$

we find, for single cells,

11 $${{{{{{{{\rm{CV}}}}}}}}}_{\lambda }^{2}={(1+{{{{{{{{\rm{CV}}}}}}}}}_{d}^{2})}^{1/4}{(1+{{{{{{{{\rm{CV}}}}}}}}}_{D}^{2})}^{1/4}-1$$

In patterning domains with many cells, CV_*λ*_ is lower due to cell averaging. The data from our simulations with *L* = 50 cell diameters precisely follows Eq. (11) up to CV_*d*,*D*_ ≈ 1, and in the case of *d* also beyond (Fig. 8E, lines), with a small proportionality constant shared by all curves. When all parameters are varied, though, CV_*λ*_ saturates at about 0.24. Larger values, such as the published CV_*λ*_ ≈ 0.4 for SHH (Fig. 4C), are unattainable even with extreme molecular variability, suggesting that the reported gradient variability^9,12^ is more technical than biological.

To examine the effect of the domain length *L*, we also varied the number of cells in the patterning domain from 20 to 200. As expected from the law of large numbers, log-fitting a variable exponential gradient over a longer domain leads to a more robustly fitted slope, such that ${{{{{{{{\rm{CV}}}}}}}}}_{\lambda } \sim 1/\sqrt{L}$ (Fig. 8F). This allows us to determine the size-dependent proportionality prefactor, resulting in

$${{{{{{{{\rm{CV}}}}}}}}}_{\lambda }^{2}=\frac{{L}_{0}}{L}\left({(1+{{{{{{{{\rm{CV}}}}}}}}}_{d}^{2})}^{1/4}{(1+{{{{{{{{\rm{CV}}}}}}}}}_{D}^{2})}^{1/4}-1\right)$$

for CV_*D*_ ≲ 1, with fit parameter *L*_0_ = 6.13 ± 0.03 μm (SE). Note that a declining CV_*λ*_ with increasing *L* is observed for the measured SHH gradient, but not for the GBS-GFP and pSMAD gradients (Fig. 7E), suggesting that amplitude effects perturbed the latter.

We further find that the fitted decay length starts to drift at moderate CV_*d*,*D*_ (Fig. 8G). If the morphogen diffusivity *D* or all parameters are noisy, *λ* is underestimated, whereas variability in the degradation rate *d* alone leads to overestimation of *λ*. This further attests to the difficulty in determining morphogen gradient parameters reliably from fitting noisy concentration profiles.

Unlike the decay length variability CV_*λ*_, the amplitude variability CV_0_ does not saturate as all cell variabilities are increased, but continues to grow linearly (Fig. 8H). We find CV_0_ to also grow mildly as the patterning domain lengthens (Fig. 8I). Finally, also the fitted amplitude is found to drift as molecular noise increases, proportionally to ${{{{{{{{\rm{CV}}}}}}}}}_{p,d,D}^{2}$ (Fig. 8J).

With these results, we can infer the physiological range of morphogen gradient variability by plugging in measured CV values. Quantitative data for the two morphogens are only available from measurements in the *Drosophila* wing disc. For Dpp, the ortholog of mouse BMP4, CV_*d*_ = 0.5 has been reported for the degradation rate, CV_*p*_ = 0.59 for the production rate, and CV_*D*_ = 0.5 for the diffusion coefficient^30^. For Hh, quantitative data is available only for the diffusion coefficient, CV_*D*_ = 0.18^29^. Measurements of other morphogens and in other species yield similar CV values^31,32^. Single cell data is available only from cell cultures. The single-cell turnover rate variability of various proteins and transcription factors in mouse embryonic stem cells has been reported to be in the range CV_*d*_ = 0.16–0.45^33^. For neural stem cell cultures only bulk measurements are available. Proteome half-life measurements yielded CV_*d*_ = 0.21 in mouse and 0.13–0.27 in human^34^. From protein half-life measurements in mouse neurons^35^, one can infer a similar degradation rate variability of CV_*d*_ = 0.35–0.5.

Overall, the physiological range of inter-cell CV values appears to be 0.1–1, but most studies report CV < 0.6, and all these values likely include some technical noise. At an intermediate value of CV_*p*,*d*,*D*_ = 0.3, the biological gradient variability is CV_*λ*_ = 0.053, CV_0_ = 0.19 at *L* = 100 μm (CV_*λ*_ = 0.027, CV_0_ = 0.20 at *L* = 400 μm), which is precise enough to explain the NKX6.1 and PAX3 domain boundary errors by opposing SHH and BMP gradients, or even by SHH alone (cf. Fig. 6C–E). Even when we use a conservative CV value of 0.6 for all three kinetic parameters, the precision of a single morphogen gradient (CV_*λ*_ = 0.062, CV_0_ = 0.39) is consistent with the NKX6.1 and PAX3 domain boundary errors (1–3 cells).

We can further use the simulations to estimate the impact of technical limitations on the measured gradient variability. The measured gradients become noisy at about 5% of the maximal value. We can represent this limitation by adding Gaussian white noise $\sim {{{{{{{\mathcal{N}}}}}}}}(0,{\eta }^{2})$ with uniform strength *η* to our simulated gradients in all cells, prior to fitting (Fig. 8K). The observed gradient variability strongly depends on the fitting method (Fig. 8L, M). Fitting the gradients in linear space (lin-fitting) always leads to elevated decay length variability, in particular for white noise exceeding 1%, but even at *η* = 0. Fitting the logarithmized gradients (log-fitting) yields significantly lower CV_*λ*_, but is insusceptible to white noise only as long as *η* ≲ 10^−5^*μ*_0_. At stronger white noise levels, we observe a power-law increase CV_*λ*_ ~ *η*^*γ*^ with exponent *γ* = 0.188 ± 0.002 (SE) and a cross-over with lin-fitting (Fig. 8L). If *η* exceeds a few percent of the amplitude, both methods yield significantly increased CV_*λ*_. Amplitude variability remains stable with lin-fitting for less than 10% white noise, whereas log-fitting yields mostly larger CV_0_ values (Fig. 8M).

In summary, our analysis suggests that natural noise in exponential morphogen gradients in the developing NT is sufficiently low to explain the previously reported progenitor domain boundary precision. Thus, both SHH and BMP gradients together—but even a single one of them alone—provide the spatial precision required to define the boundaries lying in the center of the NT with an error of only 1–3 cell diameters. But can morphogen gradients provide even higher patterning accuracy for robust development?

### Precision of progenitor domain size and progenitor number

**Fig. 9:**
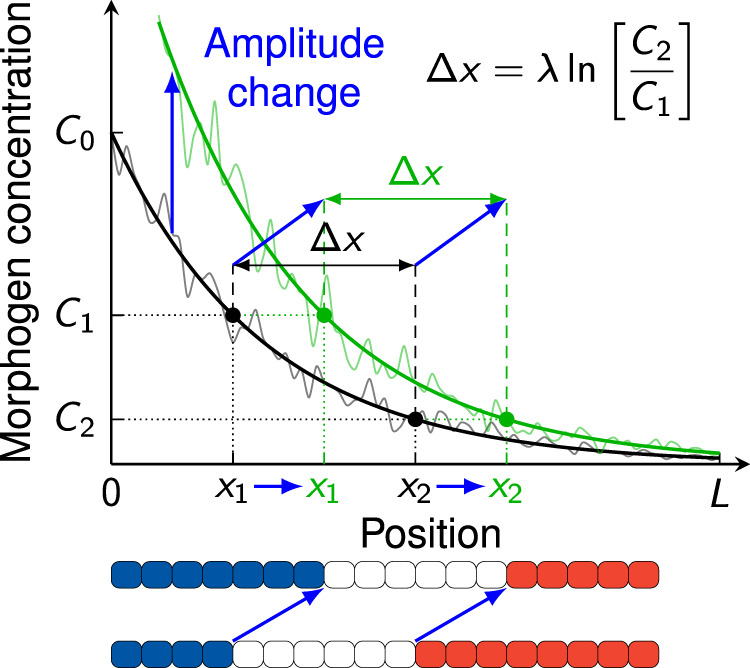
Robustness of patterning domain sizes to amplitude changes in the French flag model. The domain length Δ*x* = *x*_2_ − *x*_1_ is independent of the amplitude *C*_0_ of an exponential gradient. Amplitude changes therefore shift interior domain boundaries equally.

In the vertebrate NT, the domain boundaries define the size of the different progenitor domains, which are formed as a result of different readout thresholds, as stipulated by the French flag model^1^ (Fig. 9). Two domain boundaries located at *x*_1_ and *x*_2_ are the result of a morphogen readout at thresholds *C*_1_ = *C*(*x*_1_) and *C*_2_ = *C*(*x*_2_). As noted in^3^, the length of a domain is given by

$${{\Delta }}x={x}_{2}-{x}_{1}=\lambda \ln \left[\frac{{C}_{0}}{{C}_{2}}\right]-\lambda \ln \left[\frac{{C}_{0}}{{C}_{1}}\right]=\lambda \ln \left[\frac{{C}_{1}}{{C}_{2}}\right].$$

Notably, it is independent of the location in the entire patterning domain, and also independent of the gradient amplitude *C*_0_. Assuming that the domain width perpendicular to the *x* axis remains roughly constant along *x*, this paradigm provides a very robust mechanism to preserve the gene expression domain volume (and thus, the number of progenitor cells) during development. A change in the gradient amplitude *C*_0_ shifts both domain boundaries by the same distance, such that its size remains unchanged. The domain length is determined only by *λ*, which is stable over developmental time (Fig. 4A), and by the readout threshold ratio *C*_1_/*C*_2_. Only the very first and last progenitor domains in the pattern are affected by a transient amplitude *C*_0_, as one of their boundaries is given by the end points of the entire patterning domain, *x* = 0 and *x* = *L*.

Even in a probabilistic setting with variable gradients, the expected domain length *μ*_Δ*x*_ is unaffected by a change in amplitude, if *C*_1_, *C*_2_ and CV_0_ are constants:

12 $${\mu }_{{{\Delta }}x}={\mathbb{E}}[{{\Delta }}x]={\mu }_{\lambda }\ln \left[\frac{{C}_{1}}{{C}_{2}}\right].$$

To quantify the variability of Δ*x* in a noisy gradient, we can calculate the variance

$${\sigma }_{{{\Delta }}x}^{2}	={{{{{{\mathrm{Var}}}}}}}\,[{{\Delta }}x] ={{{{{{\mathrm{Var}}}}}}}\,[{x}_{1}]+{{{{{{\mathrm{Var}}}}}}}\,[{x}_{2}]-2{{{{{{\mathrm{Cov}}}}}}}\,[{x}_{1},{x}_{2}]\\ 	 ={\sigma }_{{x}_{1}}^{2}+{\sigma }_{{x}_{2}}^{2}+2{\mu }_{{x}_{1}}{\mu }_{{x}_{2}}-2{\mathbb{E}}\left[{x}_{1}({x}_{1}+{{\Delta }}x)\right].$$

After some elementary algebra, assuming again independence of *λ* and *C*_0_ and using Eqs. (8), (9) and (12), all terms involving the amplitude cancel out, and we find the remarkably simple form

$${\sigma }_{{{\Delta }}x}={{{{{{{{\rm{CV}}}}}}}}}_{\lambda }{\mu }_{{{\Delta }}x}={\sigma }_{\lambda }\ln \left[\frac{{C}_{1}}{{C}_{2}}\right].$$

The inaccuracy of the size of a progenitor domain therefore scales with its size itself, with CV_Δ*x*_ = CV_*λ*_ as the proportionality constant. For an exemplary domain size of *μ*_Δ*x*_ = 50 μm and a coefficient of variation CV_*λ*_ ≈ 0.05, this results in a domain size error *σ*_Δ*x*_ as low as half a cell diameter, regardless of how far away from the source the domain lies. Strikingly, unlike their spatial boundary positions, the size of the gene expression domains is completely independent of variability in the gradient amplitude. The NT patterning mechanism thus yields progenitor cell numbers with even greater accuracy than boundary locations. We emphasize that only a single morphogen gradient is required to achieve this high patterning precision.

## Discussion

High patterning precision is pivotal for robust development. We now show that the precision of morphogen gradients in the NT is far greater than previously appreciated. A single gradient—rather than the combined readout of SHH and BMP—is sufficient to generate the observed accuracy of the progenitor domain boundary positions also in the center of the mouse NT. We further show that the size of the morphogen-dependent tissue subdomains, that are not bordering the patterning domain edges, is even more precise than the individual boundary positions, because inaccuracies from amplitude variability cancel out. As a result, progenitors can be produced in more accurate numbers than previously anticipated.

These insights provide additional perspectives on gradient-based patterning and on NT development in particular. Mutual information, provided by the simultaneous readout of multiple gradients^8,36,37^, enhances patterning precision in the early Drosophila embryo^38^, and has been proposed to apply also to the mammalian NT^9,39^. While such a mechanism remains possible, given the co-regulation of central and ventral NT genes by SHH and BMP^9,40^, our finding that the boundaries of the progenitor domains in the NT can be accurately defined by a single gradient over the entire patterning period suggests a simpler patterning mechanism. This provides further prospects for tissue engineering.

The previous mathematical analysis artificially increased the reported gradient variabilities in the NT (Figs. 1 and 2), and we argue that technical limitations further inflate the reported gradient variabilities (Fig. 7). In light of the challenges in detecting exponential gradients far from the source (Fig. 3) and in measuring their variability reliably, we have developed computer simulations to estimate gradient variability based on the reported variability in morphogen production, degradation, and diffusion (Fig. 8). We find that the reported high variability of the decay length *λ* cannot arise from natural noise in these parameters alone, as it saturates at lower values than previously measured for SHH, GBS-GFP and pSMAD, at high noise levels. Our simulations confirm that for the reported molecular noise levels, the observed precision of the central progenitor boundaries is achieved even with a single gradient for the entire duration of the developmental process. Considering that the reported molecular noise levels are likely also elevated by technical errors, an even higher precision of the domain boundaries appears plausible.

Measuring the morphogen production, decay and transport rates is challenging, but still easier than the detection of low morphogen concentrations. It thus offers a complementary approach to estimating gradient variability. Current measurements of the morphogen production, decay and transport rates represent bulk measurements at the tissue level, and not yet in the mouse NT. Going forward, it will be valuable to obtain data on the single-cell variability of morphogen production and degradation rates. Currently, such data is available only from cell culture systems, but yields similar variabilities as bulk data. The reported CV values are in the range 0.1–1 across all species analyzed, including mice, flies, zebrafish, and humans. As the reported variability includes technical errors, these values present upper bounds. Even for a relatively pessimistic value of 0.6 for production, degradation and diffusion, we find that the gradient imprecision is 1–3 cells over several hundreds of micrometers, providing sufficiently accurate positional information to pattern a large domain. Local fluctuations can be reduced further through spatial and temporal averaging^5,41,42^. Moreover, in zebrafish, NT progenitor boundaries are sharpened by cell sorting^43,44^.

Due to regulatory feedback such as the up-regulation of the SHH receptor PTCH1^3,22,45^, the degradation process may not be linear, and the mean rates of morphogen production, degradation and diffusion and their variability may not be constant along the DV axis. Assuming uniform linear rates, our simulations imply that the morphogen gradients remain exponential over a wide patterning distance in the NT (Fig. 8). In case of nonlinear decay, morphogen gradients will no longer be exponential, but follow a power law^3^. It is straightforward to generalize our computational method to non-uniform rates and nonlinear processes. However, current measurement methods are not sufficiently precise to distinguish between exponential and power law gradients as the difference is observed mainly far away from the source where concentrations are low. An important question remains how cells can reliably detect very low morphogen concentrations, and what role adaptation plays in this process^7,12,20,25^.

We developed a formalism (Eq. (10)) to estimate the positional error along the entire patterning axis from the gradient variability close to the source. Gradient variabilities have been reported also for other patterning systems, including Dpp and Wg in the *Drosophila* imaginal discs^13^. Our formalism could thus be applied also in other developmental systems. We emphasize, however, that the accuracy of our formalism hinges on the accuracy of the measured gradient variabilities. Given how challenging it is to visualize morphogen gradients, technical errors are to be expected from such measurements. Based on the molecular noise simulations, we expect higher gradient precision also in other developmental systems.

More than 50 years since the publication of the French flag model^1^, it remains a matter of debate how morphogen gradients are read out^39^. Recent experiments support a threshold-based readout of the BMP gradient along the zebrafish dorsal-ventral axis^46^. Our finding that single noisy gradients provide a much more robust positional patterning mechanism than previously appreciated resolves the long-standing conundrum of how the observed patterning precision can be achieved with a simple threshold-based readout of a single gradient. This opens avenues for modelers and tissue engineers in recapitulating NT patterning in silico and in bio-engineering approaches. The presented formalism is not limited to vertebrates or flies, but applies directly also to all other morphogen-dependent patterning systems in which morphogen transport is essentially diffusive.

## Methods

### Error estimation methods

The synthetic gradients (Eq. (4)) were produced by drawing independent pairs of random parameters *λ*_*i*_ from a truncated normal and *C*_0,*i*_ from a log-normal distribution such that their statistical properties (means and standard deviations) were identical to the reported ones.

In the DEEM, the positional error is estimated directly according to Eq. (2) at a given readout threshold *C*_*θ*_ (Fig. 1D, E). We computed the set *X*_*i*_ of positions where a gradient *C*_*i*_ crosses *C*_*θ*_, using linear interpolation between discrete gradient points. For the synthetic gradients, there is only one such point (*X*_*i*_ = {*x*_*θ*,*i*_}), whereas noisy gradients can have several. For such noisy gradients, we use the middle point of the transition zone

$${x}_{\theta ,i}=\frac{\min {X}_{i}+\max {X}_{i}}{2}$$

as the readout position for each gradient *i*. Alternative averaging methods such as the arithmetic mean or median yield similar results for the data analyzed. The location and positional error follow as

$${\mu }_{x}={{{{{{\mathrm{mean}}}}}}}\,\{{x}_{\theta ,i}\}\qquad \,{{\mbox{and}}}\,\qquad {\sigma }_{x}={{{{{{\mathrm{SD}}}}}}}\,\{{x}_{\theta ,i}\},$$

taken over all embryos *i*. Repeating this procedure for different *C*_*θ*_ yields a list of (*μ*_*x*_, *σ*_*x*_) pairs.

In the NumEPM^5^ (Fig. 1D), the derivative in Eq. (3) is evaluated numerically as the slope of the mean gradient 〈*C*〉(*x*) = mean{*C*_*i*_(*x*)} (personal communication). For the synthetic (purely exponential) gradients (Eq. (4)), the derivative of the mean gradient can be directly determined from the gradient parameters as

13 $${\sigma }_{x}\approx {\left|\frac{\partial \langle C\rangle }{\partial x}\right|}^{-1}{\sigma }_{C}=\frac{n}{\mathop{\sum }\nolimits_{i = 1}^{n}{C}_{i}({x}_{\theta })/{\lambda }_{i}}{\sigma }_{C}.$$

In the FitEPM^9^ (Fig. 1D, E), a different approach is taken, in that an exponential function is lin-fitted directly (i.e., without prior log-transformation) to the mean gradient 〈*C*〉(*x*) (personal communication). This yields the parameters

$${\widetilde{C}}_{0},\widetilde{\lambda }= \mathop{{\arg}\,{\min} }\limits_{{C}_{0},\lambda }\|{{C}_{0}\exp [-x/\lambda ]-\langle C\rangle (x)\|}_{2}.$$

The slope is then estimated from the derivative of this fitted exponential:

14 $${\sigma }_{x}\approx {\left|\frac{\partial \langle C\rangle }{\partial x}\right|}^{-1}{\sigma }_{C}\approx \frac{\widetilde{\lambda }}{{\widetilde{C}}_{0}\exp \,\left[\,-\,{x}_{\theta }/\widetilde{\lambda }\right]}{\sigma }_{C}.$$

### Numerical optimization of gradient variability

We determined the gradient parameter variabilities CV_λ_ and CV_0_ in Fig. 6 by fitting Eq. (10) to the progenitor domain boundary errors with MATLAB’s nonlinear least-squares curve fitting routine lsqcurvefit.

### Inference of error bars for the positional boundary error

In Fig. 6E, we inferred the uncertainties (error bars) associated with the positional error of the domain boundaries assuming that the boundary positions are normally distributed. In this case, the standard error of the standard deviation *σ*_*x*_ is given by $\,{{\mbox{SE}}}\,[{\sigma }_{x}]\approx {\sigma }_{x}/\sqrt{2(n-1)}$ where *n* is the sample size^47^.

### Simulation of gradient variability from molecular noise

The reaction-diffusion equation was solved with MATLAB’s boundary value problem solver bvp4c with absolute and relative error tolerances of 10^−10^. At each interface between two adjacent cells, continuity of the morphogen concentration *C* and its flux − *D*∂*C*/∂*x* was imposed.

### Reporting summary

Further information on research design is available in the Nature Research Reporting Summary linked to this article.

### Supplementary information

Reporting Summary

### Source data

Source Data

## Data Availability

The individual *λ*_*i*_ and *C*_0,*i*_ for SHH (Figs. 4, 6F, G and 7B–F), the sample means and standard deviations *μ*_*λ*_, *σ*_*λ*_, *μ*_0_, *σ*_0_ for GBS-GFP and pSMAD (Figs. 1D, E and 7B–F), the positional errors of GBS-GFP, pSMAD, NKX6.1, and PAX3 (Figs. 1D, 2C–E, H–J, 6C–E and 7A), and the NT and FP/RP lengths (Figs. 2B and 3A) were extracted from the respective publications^9,12,17^. These data, together with the generated simulation data (Fig. 8E–J, L, M), are provided in the Source Data file. The individual rescaled GBS-GFP and pSMAD gradients (Figs. 2A and 3B) are not publicly available and were provided by the authors of^9^. Source data are provided with this paper.

## Code availability

The source code for Figs. 1, 2 and 8 is publicly released under the 3-clause BSD license. It is available as a git repository at https://git.bsse.ethz.ch/iber/Publications/2021_vetter_gradient_variability.
