# Supplementary material for: Precision of morphogen gradients in neural tube development
Source: Nat Commun. 2022 Mar 3;13:1145. doi: 10.1038/s41467-022-28834-3 (PMC8894346; doi:10.1038/s41467-022-28834-3)
Supplement: Supplementary file 1 — Reporting Summary [file 41467_2022_28834_MOESM1_ESM.pdf]

nature research

Corresponding author(s): Roman Vetter, Dagmar Iber

Last updated by author(s): Feb 7, 2022

Reporting Summary

Nature Research wishes to improve the reproducibility of the work that we publish. This form provides structure for consistency and transparency in reporting. For further information on Nature Research policies, see our Editorial Policies and the Editorial Policy Checklist.

Statistics

For all statistical analyses, confirm that the following items are present in the figure legend, table legend, main text, or Methods section.

n/a	Confirmed	
☐	☒	The exact sample size (n) for each experimental group/condition, given as a discrete number and unit of measurement
☐	☒	A statement on whether measurements were taken from distinct samples or whether the same sample was measured repeatedly
☒	☐	The statistical test(s) used AND whether they are one- or two-sided<br>*Only common tests should be described solely by name; describe more complex techniques in the Methods section.*
☒	☐	A description of all covariates tested
☐	☒	A description of any assumptions or corrections, such as tests of normality and adjustment for multiple comparisons
☐	☒	A full description of the statistical parameters including central tendency (e.g. means) or other basic estimates (e.g. regression coefficient) AND variation (e.g. standard deviation) or associated estimates of uncertainty (e.g. confidence intervals)
☒	☐	For null hypothesis testing, the test statistic (e.g. F, t, r) with confidence intervals, effect sizes, degrees of freedom and P value noted<br>*Give P values as exact values whenever suitable.*
☒	☐	For Bayesian analysis, information on the choice of priors and Markov chain Monte Carlo settings
☒	☐	For hierarchical and complex designs, identification of the appropriate level for tests and full reporting of outcomes
☐	☒	Estimates of effect sizes (e.g. Cohen's d, Pearson's r), indicating how they were calculated

Our web collection on statistics for biologists contains articles on many of the points above.

Software and code

Policy information about availability of computer code

Data collection	The source code is publicly released under the 3-clause BSD license. It is available as a git repository at https://git.bsse.ethz.ch/iber/Publications/2021_vetter_gradient_variability.
Data analysis	All data was processed using MATLAB (The MathWorks, Inc.) version R2020a and its Statistics and Machine Learning Toolbox. The simulation data shown in Fig. 8 were produced with the boundary value problem solver bvp4c. Model fitting in Figs. 1D-E, 2A-B, 6C-E, 8 was done using the least-squares regression tools nlinfit, fitnlm and lsqcurvefit.

For manuscripts utilizing custom algorithms or software that are central to the research but not yet described in published literature, software must be made available to editors and reviewers. We strongly encourage code deposition in a community repository (e.g. GitHub). See the Nature Research guidelines for submitting code & software for further information.

Data

Policy information about availability of data

All manuscripts must include a data availability statement. This statement should provide the following information, where applicable:

- Accession codes, unique identifiers, or web links for publicly available datasets
- A list of figures that have associated raw data
- A description of any restrictions on data availability

The individual λ_i and C_0,i for SHH (Figs. 4,6F-G,7B-F), the sample means and standard deviations μ_λ, σ_λ, μ_0, σ_0 for GBS-GFP and pSMAD (Figs. 1D-E,7B-F), the positional errors of GBS-GFP, pSMAD, NKX6.1, and PAX3 (Figs. 1D,2C-E,H-J,6C-E,7A), and the NT and FP/RP lengths (Figs. 2B,3A) were extracted from the respective publications [Kicheva et al., 2014; Cohen et al., 2015; Zagorski et al., 2017]. These data, together with the generated simulation data (Fig. 8E-J,L,M), are provided in the Source Data file.
The individual rescaled GBS-GFP and pSMAD gradients (Figs. 2A,3B) are not publicly available and were provided by the authors of [Zagorski et al., 2017]. They can

be requested from James Briscoe.

Field-specific reporting

Please select the one below that is the best fit for your research. If you are not sure, read the appropriate sections before making your selection.

- [x] Life sciences
- [ ] Behavioural & social sciences
- [ ] Ecological, evolutionary & environmental sciences

For a reference copy of the document with all sections, see nature.com/documents/nr-reporting-summary-flat.pdf

Life sciences study design

All studies must disclose on these points even when the disclosure is negative.

Sample size	This work does not include new experiments. For the numerical simulations, we chose 1000 independent random realizations as a good balance between manageable computational effort and high numerical precision (yielding small standard errors as shown in the plots).
Data exclusions	No data were excluded.
Replication	This work does not include new experiments. The simulations are fully reproducible from scratch with the freely available standalone source code.
Randomization	This work does not include new experiments or dataset grouping. The simulations use statistically independent realizations - no grouping or correlations involved.
Blinding	This work does not include any new group allocation, hence blinding does not apply.

Reporting for specific materials, systems and methods

We require information from authors about some types of materials, experimental systems and methods used in many studies. Here, indicate whether each material, system or method listed is relevant to your study. If you are not sure if a list item applies to your research, read the appropriate section before selecting a response.

Materials & experimental systems

n/a	Involved in the study	
x		Antibodies
x		Eukaryotic cell lines
x		Palaeontology and archaeology
x		Animals and other organisms
x		Human research participants
x		Clinical data
x		Dual use research of concern

Methods

n/a	Involved in the study	
x		ChIP-seq
x		Flow cytometry
x		MRI-based neuroimaging
